# Integrative Approach to Quality Assessment of Medical Journals Using Impact Factor, Eigenfactor, and Article Influence Scores

**DOI:** 10.1371/journal.pone.0010204

**Published:** 2010-04-15

**Authors:** Jacques Rizkallah, Don D. Sin

**Affiliations:** Department of Medicine (Respiratory Division) and the Providence Heart and Lung Institute at St. Paul's Hospital, Vancouver, British Columbia, Canada; Indiana University - Bloomington, United States of America

## Abstract

**Background:**

Impact factor (IF) is a commonly used surrogate for assessing the scientific quality of journals and articles. There is growing discontent in the medical community with the use of this quality assessment tool because of its many inherent limitations. To help address such concerns, Eigenfactor (ES) and Article Influence scores (AIS) have been devised to assess scientific impact of journals. The principal aim was to compare the temporal trends in IF, ES, and AIS on the rank order of leading medical journals over time.

**Methods:**

The 2001 to 2008 IF, ES, AIS, and number of citable items (CI) of 35 leading medical journals were collected from the Institute of Scientific Information (ISI) and the http://www.eigenfactor.org databases. The journals were ranked based on the published 2008 ES, AIS, and IF scores. Temporal score trends and variations were analyzed.

**Results:**

In general, the AIS and IF values provided similar rank orders. Using ES values resulted in large changes in the rank orders with higher ranking being assigned to journals that publish a large volume of articles. Since 2001, the IF and AIS of most journals increased significantly; however the ES increased in only 51% of the journals in the analysis. Conversely, 26% of journals experienced a downward trend in their ES, while the rest experienced no significant changes (23%). This discordance between temporal trends in IF and ES was largely driven by temporal changes in the number of CI published by the journals.

**Conclusion:**

The rank order of medical journals changes depending on whether IF, AIS or ES is used. All of these metrics are sensitive to the number of citable items published by journals. Consumers should thus consider all of these metrics rather than just IF alone in assessing the influence and importance of medical journals in their respective disciplines.

## Introduction

The impact factor (IF), which is a score calculated each year by the Institute for Scientific Information (ISI), is widely considered as one of the leading proxies for evaluating the quality, importance, and influence of medical journals to their respective discipline (Science Citation Index, Journal Citation Report. Institute for Scientific Information, www.isinet.com). [1] Medical editors frequently use the IF as a performance index of their journal and a means of ranking their journals relative to their peers.[2], [3], [4], [5] Some journals use the IF to “advertise” their quality and to entice potential authors in submitting high-quality papers to them. Promotion committees of academic institutions commonly use the IF to judge the quality of publications of applicants for promotion and tenure and departmental chairs may use it in the hiring and assessment process of new recruits. [6] Increasingly, however, there is growing discontent with the IF as a tool for determining “quality” and “prestige” of journals [7], [8]. One reason is that the distribution of citations is non-parametric with fewer than 20% of the articles accounting for more than 50% of the total number of citations of journals and with many articles that never receive any citations [9], [10]. Moreover, IF only counts the number of citations without taking into account the source of the citations (ie. citations from prestigious journals are worth no more than citations from lower-tier journals) or makes any allowances for the “citation culture” between journals and across disciplines [7]. It is also now well recognized that journal's IF can be increased by reducing the number of original research papers and increasing the number of editorials (which are not counted in the denominator of IF), review papers, which receive on average twice as many citations as original articles [9], [11] and by encouraging self-citations [7], [11]. Original research papers, however, are the main “engines” of generating new knowledge and, by decreasing their publication rate, journals may be mitigating dissemination of scientific knowledge and curtailing scientific discourse. Over time, this may increase the IF but paradoxically reduce the overall influence of these journals on the scientific community as fewer scientists and clinicians read the journal. To address these and other concerns with the IF, other instruments including those that take into account the quality as well as the quantity of citations, have been proposed [12], [13], [14]. This concept was first proposed by Pinski and Narin [15], who suggested that journals should be ranked according to their eigenvector centrality in a citation network. With the recent success of Google's ranking system for web pages, this concept has been modified to include algorithms based on a PageRank system [13]. Although there are several different algorithms in use, the two that have gained the most attention in recent years are Scimago Journal Rank (SJR) (http://www.scimagojr.com/index.php) and Eigenfactor score (ES) (http://eigenfactor.org/), both of which use an iterative weighting system to calculate a summary index that reflects both the “quality” and the “quantity” of citations received by these journals based on a PageRank algorithm [12], [15]. Despite the differences in the way in which weight-based and non-weight based methods are derived, studies have shown that in any given year, scores based on a PageRank algorithm correlate well with those based on traditional IF and produce similar rank order of medical journals [14], [16]. However, it is not known whether the temporal trends in these scores produce similar or differential rank orders of these journals. Since ES is at least in part dependent on the number of citable items published by journals in any given year [17], [18], by reducing the publication rate, it is possible for a journal to increase IF without changing its ES (and vice versa). Thus, the primary aim of the present study was to determine the changes in IF and ES across the major general and sub-specialty medical journals over the past 8 years.

## Methods

### Selection of Journals

We decided *a priori* to evaluate the temporal trends in the impact factor (IF) and Eigenfactor Score (ES) in 35 general and subspecialty clinical journals between 2001 and 2008. We chose this timeframe to mitigate the influence of name changes of journals in the IF and ES calculations and to ensure comparability of data across the journals. To ensure reasonable representation of journals from each discipline, we chose the three mostly highly ranked journals per discipline as determined by the 2008 IF except for respiratory medicine and endocrinology in which four rather than three journals were selected. We did this to mitigate the potential effect of overlap of content and audience of journals in the “respiratory system” and “critical care medicine” (e.g. the *American Journal of Respiratory and Critical Medicine* is listed both categories) and to ensure that there is adequate representation of non-diabetic papers (and audience) in “endocrinology” as the top two journals under this category were diabetes-focused (e.g. *Diabetes* and *Diabetes Care*). From the Thompson Reuters' Journal Citation Reports (http://admin-apps.isiknowledge.com/JCR/JCR?PointOfEntry=Home&SID=3EIG4M34Amad6eKDPA) and the Eigenfactor.org websites (http://eigenfactor.org/), two independent reviewers (JR, DS) abstracted data on the IF, ES, citable items and Article Influence Score (AIS) on these journals. The data were imported into an Excel Spreadsheet and any disagreements were resolved by iteration and consensus.

The journals that were evaluated included *Annals of Neurology (Ann Neurol), Annals of the Rheumatic Diseases (Ann Rheum Dis), Arthritis and Rheumatism (Art Rheum/Ar C Res), Brain, Circulation, Clinical Infectious Diseases (Clin Inf Dis), Diabetes, Diabetes Care, European Heart Journal (Eur Heart J), Gastroenterology, Gut, Hepatology, Intensive Care Medicine (Intens Care Med), Journal of the American Medical Association (JAMA), Journal of American College of Cardiology (J Am Coll Cardiol), Journal of Bone and Mineral Research (J Bone Miner Res); Journal of Clinical Endocrinology and Metabolism (J Clin Endocr Metab), Journal of Clinical Oncology (J Clin Oncol), Journal of Infectious Diseases (J Infect Dis), Journal of the National Cancer Institute (J Natl Cancer I), Journal of Neurosciences (J Neurosci), Lancet, Lancet Infectious Diseases (Lancet Infect Dis), Lancet Oncology (Lancet Oncol), New England Journal of Medicine (N Engl J Med), Rheumatology, Allergy, American Journal of Respiratory and Critical Care Medicine (Am J Respir Crit Care Med), Clinical and Experimental Allergy (Clin Exp Allergy), Chest, Critical Care, Critical Care Medicine (Crit Care Med), European Respiratory Journal (Eur Resp J), Journal of Allergy and Clinical Immunology (J Allergy Clin Immunology)*, and *Thorax*. We did not include any non-clinical journals.

### Impact Factor

The IF is published by ISI each year for all indexed journals and is calculated based on a three-year period. It reflects the average number of times that papers are cited up to two years following publication. For example, the 2009 IF for a journal would be calculated by taking the number of times articles (original, reviews, proceedings or notes) published in 2007 and 2008 were cited in 2009 and dividing this number by the total number of articles, reviews, proceedings, guidelines or consensus statements that were published in this journal in 2007 and 2008. Editorials and letters to the editors are generally excluded from the denominator but can be counted in the numerator of the impact factor. In general, review articles, consensus statements and clinical guidelines are cited more frequently than original articles[19].

### Eigenfactor Score (ES)

For each of these journals, we retrieved data on the ES from http://www.eigenfactor.org. ES is calculated based on a complex algorithm that takes into account not only the quantity of citations but also their “quality” by assigning weights to the source of the citations. The full details of the algorithm can be found at http://www.eigenfactor.org/methods.htm. In brief, the algorithm assigns quality scores to journals by creating a citation network in which journal articles are first randomly selected. The citation lists from these retrieved articles are then used by the network to select the next set of journals. The citation lists from this batch of journals are then used by the network to select the third set of journals. This process continues indefinitely creating a hierarchical ranking of journals based on the frequency of citations. The network assumes that journals that are highly cited are to be of high quality, while those that are infrequently cited are deemed to be of lower quality. Importantly, the ES has no denominator. Thus, journals that publish a lot of articles have higher ES than those that publish very few articles if the average quality of the published articles is similar between these journals.

### Article Influence Score (AIS)

Article Influence™ Score (AIS) is derived from ES and conceptually similar to IF in that there is a numerator as well as a denominator (i.e. number of citable papers) except that it uses ES (rather than the total number of citations) as the numerator. Thus, dissimilar to IF where all citations are counted equally regardless of their source, in AIS, each citation is multiplied by the “quality” of the citing journals, resulting in greater weights for citations that come from highly cited journals, and less weight to poorly cited journals. To facilitate interpretation, the AIS is normalized, so that the mean article in the Journal of Citation Reports® has an AIS of 1.00.

### Statistical Analysis

The journals were ranked based on the published 2008 ES, AIS, and IF scores. We also retrieved the 2001 to 2008 ES, AIS, IF scores, and number of citable items (CI) in order to determine the temporal trends in these values. The statistical significance of the temporal trends was determined using a chi-square test for trend. A p-value of less than 0.05 was considered statistically significant. All analyses were conducted using SAS version 9.1 (Carey, N.C.).

## Results

### 2008 AIS, IF, and ES

The 2008 ES, AIS, and IF values of selected medical journals are shown in Table 1. Of the evaluated journals, the overall leader was the *New England Journal of Medicine* irrespective of the metric used to measure quality. However, the rankings for the remaining journals changed depending on the score that was used. For instance, using the traditional IF score, the 2^nd^ leading journal in 2008 was *JAMA*, followed by the *Lancet, J Clin Oncol* and *J Natl Cancer I*. In general, the AIS and IF values provided similar rank orders, with few notable exceptions including *J Neurosci*, which was ranked 11^th^ based on AIS and 20^th^ based on IF and *J Allergy Clin Immunol*, which was ranked 20^th^ based on AIS and 14^th^ based on IF.

**Table 1:** Journal Rankings Based On Their 2008 Eigenfactor, Impact Factor and the Article Influence Score.

Journal	Eigenfactor Score	Article Influence Score	Impact Factor
N Engl J Med	0.68060 (1)	18.764 (1)	50.017 (1)
J Neurosci	0.52199 (2)	3.544 (11)	7.452 (20)
Circulation	0.48312 (3)	4.794 (5)	14.595 (6)
Lancet	0.41221 (4)	9.953 (3)	28.409 (3)
JAMA	0.38132 (5)	11.153 (2)	31.718 (2)
J Clin Oncol	0.34752 (6)	4.164 (7)	17.157 (4)
J Am Coll Cardiol	0.22767 (7)	3.727 (10)	11.438 (10)
J Clin Endocr Metab	0.16459 (8)	1.803 (27)	6.325 (27)
Gastroenterology	0.15356 (9)	3.913 (9)	12.591 (9)
Diabetes	0.14843 (10)	2.989 (17)	8.398 (18)
Diabetes Care	0.13757 (11)	2.508 (19)	7.349 (21)
Clin Inf Dis	0.13735 (12)	2.345 (21)	8.266 (19)
Art Rheum/Ar C Res	0.12799 (13)	2.062 (25)	6.787 (24)
J Infect Dis	0.12043 (14)	2.064 (24)	5.682 (29)
Am J Resp Crit Care Med	0.11875 (15)	3.125 (15)	9.792 (13)
Hepatology	0.11722 (16)	3.363 (13)	11.355 (11)
Chest	0.11173 (17)	1.516 (30)	5.154 (31)
J Natl Cancer I	0.09924 (18)	5.791 (4)	14.933 (5)
Brain	0.09868 (19)	3.527 (12)	9.603 (16)
Eur Heart J	0.09764 (20)	3.010 (16)	8.917 (17)
J Allergy Clin Immunol	0.09561 (21)	2.488 (20)	9.773 (14)
Crit Care Med	0.08298 (22)	1.852 (26)	6.594 (25)
Ann Neurol	0.08000 (23)	3.338 (14)	9.935 (12)
Gut	0.07273 (24)	2.551 (18)	9.766 (15)
Eur Resp J	0.06172 (25)	1.775 (29)	5.545 (30)
J Bone Miner Res	0.06006 (26)	2.295 (22)	6.443 (26)
Ann Rheum Dis	0.05717 (27)	1.802 (28)	7.188 (22)
Thorax	0.04291 (28)	2.280 (23)	7.069 (23)
Intens Care Med	0.03599 (29)	1.214 (33)	5.055 (32)
Lancet Oncol	0.03510 (30)	3.947 (8)	13.283 (7)
Rheumatology	0.03438 (31)	1.188 (34)	4.136 (34)
Lancet Infect Dis	0.03202 (32)	4.689 (6)	13.165 (8)
Allergy	0.02893 (33)	1.355 (31)	6.204 (28)
Clin Exp Allergy	0.02779 (34)	1.150 (35)	3.556 (35)
Critical Care	0.02355 (35)	1.346 (32)	4.553 (33)

Data presented as value (column rank).

**Figure 1:**
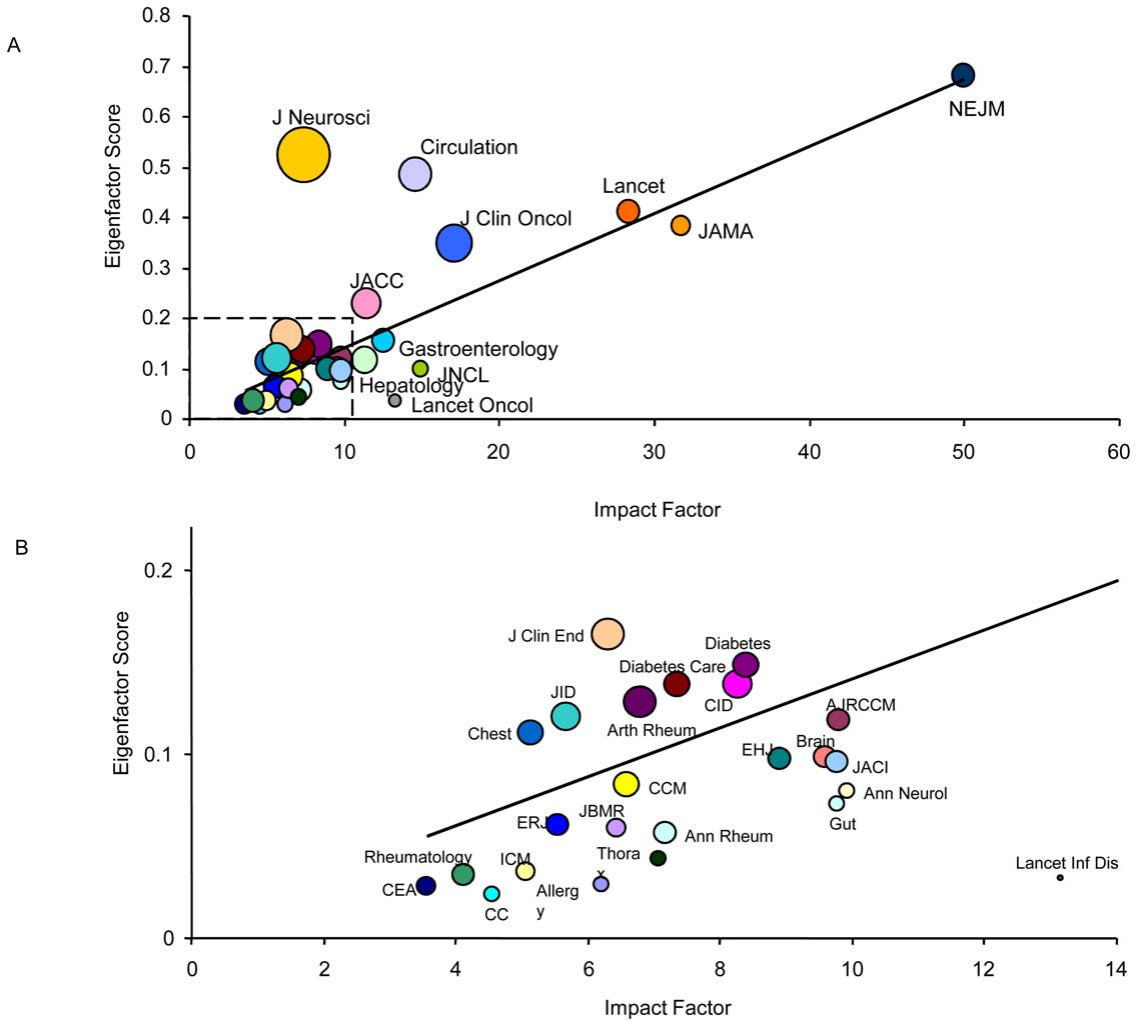
The Relationship Between Impact Factor (IF) and Eigenvalue Score (ES) In 2008. The area of the circles is proportional to the number of citable items published in 2008. The area of the dotted line is expanded in figure 1B. R^2^ = 0.5721; p<0.0001.

Using ES values resulted in large changes in the rank order of the selected journals. While the *N Engl J Med* retained the top spot, *J Neurosci* took over 2^nd^ spot on the list, followed by *Circulation, Lancet* and *JAMA*. In general, journals that published a lot of papers had higher ES values than journals that published small volumes of papers (figure 1). For instance, *Lancet Oncol*, which was ranked 8^th^ on the AIS and 7^th^ on the IF lists, was ranked only 30^th^ on the ES list. On the other hand, *J Clin Endocr Metab*, which ranked 27^th^ on both the AIS and IF lists, was ranked 8^th^ on the ES list.

Both the IF and CI correlated significantly with the 2008 ES values (p value for both <.0001). The partial square value for IF was 0.5721 and that for CI was 0.3678. Thus, collectively, they accounted for 94% of the variance in the 2008 ES values. As the IF values increased so did the ES values (See figure 1A and 1B). In general, however, journals with a high number of citable items displayed higher ES values than those that had a small number of citable items.

### Trends in IF, ES, AIS, and CI Between 2001 and 2008

**Figure 2:**
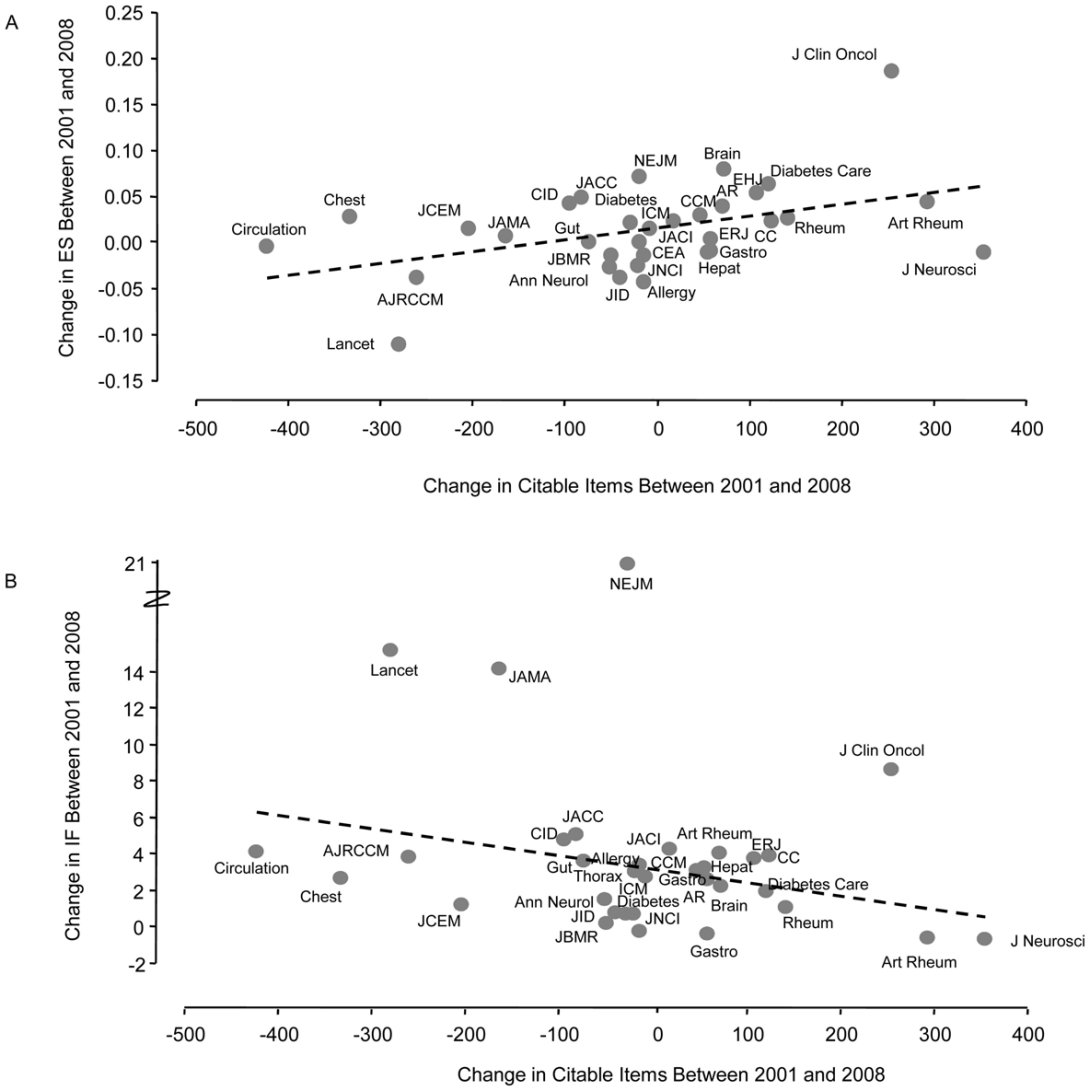
The Relationship of Changes in Citable Items Between 2001 and 2008 to Changes in Eigenfactor Score and Impact Factor Between 2001 and 2008. R^2^ = 0.1957; p = 0.0099 for the relationship between changes in citable items and changes in Eigenfactor score and R^2^ = 0.1216; p = 0.0505* for the relationship between changes in citable items and changes in the impact factor. *The *New England Journal of Medicine* was excluded from the regression analysis, as it was an extreme outlier.

Since 2001, the IF of 77% (27/35) of the journals included this analysis increased significantly (Table 2). Only *J Neurosci* experienced a significant decline in IF. In the remaining journals, the IF did not change significantly over time. In contrast, only 51% ( = 18/35) of the journals increased their ES values over the 8 years, while 26% ( = 9/35) of the journals experienced a decline in their EF values (table 3). The discordance between the temporal trends in IF and ES was largely driven by the temporal changes in the number of citable items published by each of the journals (see figure 2A). In 20% of the journals, the number of citable items increased and in another 20% the number of citable items decreased over time. In the remaining 60%, the number of citable items did not change significantly (Table 4; figure 2A). In general, as the number of citable items decreased, the IF of the journals increased, though this relationship did not reach statistical significance (p = 0.132) largely due to the extreme effects of the *New England Journal of Medicine*, whose IF score increased by 21 in the absence of any significant changes in the number of citable items over the 8 years of the study. The removal of the *New England Journal of Medicine* from this analysis, however, led to a significant relationship between the temporal trends in CI and IF (figure 2B; p = 0.05). There were journals whose IF score and the number citable items both increased during this period of time (see Tables 2 and 4). These included *the European Heart Journal*, *Brain*, *Rheumatology*, and *Critical Care*. On the other hand, journals such as *Intensive Care Medicine*, the *Journal of American College of Cardiology*, *Diabetes Care*, the *Journal of Infectious Diseases, Hepatology*, *Annals of Rheumatic Diseases, Chest, Allergy*, the *European Respiratory Journal, Critical Care Medicine, Lancet Oncology, Clinical Infectious Diseases,* the *Journal of Clinical Oncology, Lancet Infectious Diseases*, the *Journal of Allergy and Clinical Immunology, and the New England Journal of Medicine* increased their IF without significantly changing the number of citable items that were published per year. Conversely, a few journals such as the *Lancet, Circulation, the American Journal of Respiratory and Critical Care Medicine, the Journal of the American Medical Association, Gut, and Thorax* increased their IF but at the same time decreased the number of citable items published per year. Interestingly, some journals such as the *Journal of Bone and Mineral Research* and *Journal of National Cancer Institute* reduced the number of citable items without experiencing an increase in their IF. The temporal trends in AIS were similar to those of IF. 66% of the journals experienced an increase in AIS, while 6% experienced a decline (Table 5).

**Table 2:** Temporal Trends in the Impact Factor of Common Medical Journals Between 2001 and 2008.

Journal	2001	2002	2003	2004	2005	2006	2007	2008	P trend
Allergy	2.852	3.666	3.161	3.496	4.120	5.334	5.014	6.204	0.0006↑
Am J Resp Crit Care Med	5.956	6.567	8.876	8.123	8.689	9.091	9.074	9.792	0.0083↑
Ann Neurol	8.481	8.603	7.717	8.097	7.571	8.051	8.813	9.935	0.2575
Ann Rheum Dis	3.188	3.593	3.827	3.916	6.956	5.767	6.411	7.188	0.0021↑
Art Rheum/Ar C Res	7.389	7.379	7.190	7.414	7.421	7.751	7.677	6.787	0.8108
Brain	7.407	7.122	7.967	8.201	7.535	7.617	8.568	9.603	0.0265↑
Chest	2.480	2.969	3.264	3.118	4.008	3.924	4.143	5.154	0.0008↑
Circulation	10.517	10.255	11.164	12.563	11.632	10.940	12.755	14.595	0.0174↑
Clin Exp Allergy	3.826	3.721	3.176	3.069	3.553	3.668	3.729	3.556	0.9845
Clin Inf Dis	3.545	4.750	5.393	5.594	6.510	6.186	6.750	8.266	0.0002↑
Crit Care Med	3.486	3.361	4.195	4.182	5.077	6.599	6.283	6.594	0.0003↑
Critical Care	0.701	0.876	1.911	3.214	2.932	3.116	3.834	4.553	0.0002↑
Diabetes	7.700	8.256	8.298	8.848	8.028	7.955	8.261	8.398	0.5307
Diabetes Care	5.404	5.477	7.501	7.071	7.844	7.912	7.851	7.349	0.0239↑
Eur Heart J	5.153	6.131	5.997	6.247	7.341	7.286	7.924	8.917	<.0001↑
Eur Resp J	2.989	2.931	2.999	3.096	3.947	5.076	5.349	5.545	0.0006↑
Gastroenterology	13.020	13.440	12.718	13.092	12.386	12.457	11.673	12.591	0.0427↓
Gut	6.170	6.323	5.883	6.601	7.692	9.002	10.015	9.766	0.0008↑
Hepatology	8.096	9.825	9.503	10.416	9.792	10.446	10.734	11.355	0.0038↑
Intens Care Med	2.314	2.041	2.971	3.304	3.724	4.406	4.623	5.055	<.0001↑
J Allergy Clin Immunol	5.506	6.282	6.831	7.205	7.667	8.829	8.115	9.773	0.0001↑
J Am Coll Cardiol	6.374	6.278	7.599	9.133	9.200	9.701	11.054	11.438	<.0001↑
J Bone Miner Res	6.230	6.329	6.225	5.436	6.527	6.635	6.004	6.443	0.6879
J Clin Endocr Metab	5.160	5.199	5.873	5.778	6.020	5.799	5.493	6.325	0.0523
J Clin Oncol	8.530	9.868	10.864	9.835	11.810	13.598	15.484	17.157	0.0002↑
J Infect Dis	4.910	4.857	4.481	4.943	4.953	5.363	6.035	5.682	0.0158↑
J Natl Cancer I	14.240	14.500	13.844	13.856	15.171	15.271	15.678	14.933	0.0538
J Neurosci	8.178	8.045	8.306	7.907	7.506	7.453	7.490	7.452	0.0037↓
JAMA	17.569	16.586	21.455	24.831	23.494	23.175	25.547	31.718	0.0021↑
Lancet	13.251	15.397	18.316	21.713	23.878	25.800	28.638	28.409	<.0001↑
Lancet Infect Dis	n/a§	n/a§	n/a§	10.788	10.008	11.808	12.058	13.165	0.0002↑
Lancet Oncol	n/a§	n/a§	7.411	8.794	9.608	10.119	12.247	13.283	0.0003↑
N Engl J Med	29.065	31.736	34.833	38.570	44.016	51.296	52.589	50.017	0.0001↑
Rheumatology	3.062	3.251	3.760	4.102	4.226	4.052	4.045	4.136	0.0109↑
Thorax	4.090	4.078	4.188	5.040	6.150	6.064	6.226	7.069	0.0002↑

Significant increase over time denoted by (↑), while significant decrease is denoted (↓).

§ n/a  =  not available.

**Table 3:** Temporal Trends in Eigenfactor Score of Common Medical Journals Between 2001 and 2008.

Journal	2001	2002	2003	2004	2005	2006	2007	2008	P trend
Allergy	0.07282	0.080834	0.075831	0.076492	0.078997	0.085935	0.02562	0.02893	0.0876
Am J Resp Crit Care Med	0.15806	0.17425	0.17693	0.16138	0.15427	0.14639	0.12449	0.11875	0.0065↓
Ann Neurol	0.10808	0.10385	0.10094	0.098948	0.091732	0.092764	0.08541	0.08000	<.0001↓
Ann Rheum Dis	0.018389	0.019886	0.024583	0.030932	0.040236	0.046286	0.05478	0.05717	<.0001↑
Art Rheum/Ar C Res	0.08375	0.08367	0.09978	0.1111	0.1227	0.12685	0.13066	0.12799	0.0003↑
Brain	0.01939	0.016637	0.017696	0.01884	0.0155	0.015057	0.09959	0.09868	0.0373↑
Chest	0.084284	0.089413	0.097728	0.098228	0.10029	0.1102	0.10961	0.11173	<.0001↑
Circulation	0.48859	0.50912	0.5136	0.55557	0.55648	0.54769	0.53421	0.48312	0.6521
Clin Exp Allergy	0.041638	0.033118	0.032557	0.029458	0.031495	0.029831	0.0284	0.02779	0.0040↓
Clin Inf Dis	0.09477	0.10738	0.11542	0.11965	0.14562	0.14695	0.13481	0.13735	0.0065↑
Crit Care Med	0.053328	0.059861	0.06925	0.07191	0.075306	0.074332	0.07932	0.08298	0.0002↑
Critical Care	0.000450	0.000930	0.001826	0.003928	0.005918	0.009211	0.01766	0.02355	0.0163↑
Diabetes	0.12751	0.13088	0.14085	0.15923	0.15884	0.16705	0.16425	0.14843	0.0314↑
Diabetes Care	0.0747	0.088212	0.088662	0.09683	0.11157	0.12342	0.13564	0.13757	<.0001↑
Eur Heart J	0.043836	0.048415	0.049693	0.0486	0.059156	0.070785	0.08836	0.09764	0.0007↑
Eur Resp J	0.058554	0.057278	0.051405	0.051253	0.052220	0.062227	0.06297	0.06172	0.2168
Gastroenterology	0.1629	0.16054	0.15203	0.14565	0.15427	0.15842	0.15861	0.15356	0.5501
Gut	0.071954	0.069639	0.070522	0.072297	0.070967	0.077763	0.08003	0.07273	0.1039
Hepatology	0.12885	0.13153	0.13122	0.12194	0.11457	0.11437	0.10952	0.11722	0.0071↓
Intens Care Med	0.021169	0.020998	0.026719	0.026049	0.032777	0.030637	0.03164	0.03599	0.0005↑
J Allergy Clin Immunol	0.07282	0.080834	0.075831	0.076492	0.078997	0.085935	0.08087	0.09561	0.0203↑
J Am Coll Cardiol	0.17946	0.17849	0.19083	0.20115	0.20778	0.22425	0.24561	0.22767	0.0005↑
J Bone Miner Res	0.074053	0.074098	0.071383	0.063249	0.069853	0.064298	0.05845	0.06006	0.0026↓
J Clin Endocr Metab	0.14938	0.15631	0.17443	0.18105	0.19196	0.19941	0.17641	0.16459	0.2057
J Clin Oncol	0.16178	0.1829	0.21401	0.22286	0.2357	0.28292	0.32292	0.34752	<.0001↑
J Infect Dis	0.15884	0.15267	0.14252	0.14191	0.14062	0.14092	0.12948	0.12043	0.0004↓
J Natl Cancer I	0.12546	0.12639	0.12517	0.11642	0.11327	0.10658	0.10638	0.09924	<.0001↓
J Neurosci	0.53382	0.54054	0.53744	0.5374	0.53002	0.50843	0.48824	0.52199	0.0495↓
JAMA	0.37474	0.39978	0.43187	0.44731	0.44905	0.45493	0.41748	0.38132	0.6510
Lancet	0.52281	0.53936	0.5347	0.54342	0.51354	0.5002	0.45171	0.41221	0.0093↓
Lancet Infect Dis	n/a§	n/a§	n/a§	0.015212	0.021729	0.028966	0.03367	0.03202	0.0204↑
Lancet Oncol	n/a§	n/a§	0.008829	0.015841	0.01954	0.023433	0.03318	0.03510	0.0002↑
N Engl J Med	0.6099	0.63452	0.65826	0.69058	0.68049	0.7183	0.69405	0.68060	0.0165↑
Rheumatology	0.0085104	0.0086894	0.0079648	0.025789	0.012345	0.01346	0.03424	0.03438	0.0191↑
Thorax	0.042666	0.043209	0.039287	0.04243	0.042654	0.046807	0.04395	0.04291	0.3280

§ n/a  =  not available.

Significant increase over time denoted by (↑), while significant decrease is denoted (↓).

**Table 4:** Temporal Trends in Citable Items of Common Medical Journals Between 2001 and 2008.

Journal	2001	2002	2003	2004	2005	2006	2007	2008	P trend
Allergy	201	207	184	175	211	205	188	188	0.6392
Am J Resp Crit Care Med	569	446	380	330	375	330	299	309	0.0059↓
Ann Neurol	220	232	255	237	251	204	134	169	0.0626
Ann Rheum Dis	230	283	339	377	364	290	295	302	0.5571
Art Rheum/Ar C Res	346	478	513	597	505	559	626	640	0.0053↑
Brain	202	196	227	235	256	276	268	275	0.0003↑
Chest	720	729	704	654	915	492	560	388	0.0912
Circulation	1029	995	1084	1129	980	682	670	607	0.0108↑
Clin Exp Allergy	215	239	226	264	215	180	205	201	0.1856
Clin Inf Dis	611	513	572	431	564	490	425	517	0.1732
Crit Care Med	397	474	476	363	313	468	379	445	0.8089
Critical Care	52	59	66	125	140	116	176	177	0.0005↑
Diabetes	420	556	405	497	478	481	379	391	0.3059
Diabetes Care	301	99	467	474	482	460	591	423	0.0850
Eur Heart J	187	186	211	250	312	360	330	296	0.0060↑
Eur Resp J	284	403	402	294	285	306	285	343	0.5201
Gastroenterology	295	361	316	376	349	361	368	353	0.1338
Gut	266	352	307	280	243	223	255	193	0.0292↓
Hepatology	329	363	296	319	284	318	355	384	0.4380
Intens Care Med	267	163	325	294	213	246	259	260	0.8746
J Allergy Clin Immunol	329	294	325	348	169	338	350	347	0.7633
J Am Coll Cardiol	543	529	506	591	561	591	506	462	0.4743
J Bone Miner Res	261	259	262	208	229	232	229	212	0.0305↓
J Clin Endocr Metab	867	813	850	868	950	741	709	664	0.0712
J Clin Oncol	511	554	643	595	1021	734	707	766	0.1221
J Infect Dis	533	585	548	581	561	440	502	494	0.1091
J Natl Cancer I	165	156	157	159	158	156	143	145	0.0103↓
J Neurosci	1083	1194	1288	1233	1232	1415	1476	1438	0.0016↑
JAMA	389	383	377	351	380	267	229	225	0.0025↓
Lancet	569	522	553	415	360	301	305	289	0.0003↓
Lancet Infect Dis	n/a§	n/a§	n/a§	58	58	62	65	50	0.6821
Lancet Oncol	n/a§	n/a§	94	89	87	91	93	105	0.2091
N Engl J Med	375	378	366	316	308	303	343	356	0.2251
Rheumatology	177	190	221	240	264	275	305	320	<.0001↑
Thorax	182	207	198	195	165	163	155	163	0.0210↓

§ n/a  =  not available.

Significant increase over time denoted by (↑), while significant decrease is denoted (↓).

**Table 5:** Temporal Trends in Article Influence Scores of Common Medical Journals Between 2001 and 2008.

Journal	2001	2002	2003	2004	2005	2006	2007	2008	P trend
Allergy	1.4877	1.6964	1.6234	1.7028	1.9319	2.1341	1.122	1.355	0.6576
Am J Resp Crit Care Med	1.8261	2.0711	2.2495	2.2829	2.4995	2.8246	2.877	3.125	<.0001↑
Ann Neurol	3.3780	3.2923	3.2187	3.2531	3.1068	3.1693	3.116	3.338	0.2938
Ann Rheum Dis	0.7720	0.80928	0.89922	0.98872	1.357	1.3905	1.660	1.802	<.0001↑
Art Rheum/Ar C Res	1.9775	1.8535	4.2411	3.0898	2.4768	2.0633	2.119	2.062	0.6555
Brain	3.2601	3.1851	3.5698	3.458	3.601	3.4876	3.6	3.527	0.0311↑
Chest	0.94489	0.98896	1.0677	1.0967	1.1732	1.2089	1.349	1.516	<.0001↑
Circulation	3.9945	4.018	4.0969	4.3223	4.2973	4.273	4.703	4.794	0.0007↑
Clin Exp Allergy	0.9400	0.78927	0.80948	0.7639	0.86069	0.84054	1.087	1.150	0.0853
Clin Inf Dis	1.1824	1.3578	1.5493	1.6781	2.1362	2.117	2.137	2.345	<.0001↑
Crit Care Med	1.8261	2.0711	2.2495	2.2829	2.4995	2.8246	1.612	1.852	0.9762
Critical Care	1.8261	2.0711	2.2495	2.2829	2.4995	2.8246	1.129	1.346	0.4508
Diabetes	2.9060	2.8689	2.7892	3.0744	2.953	2.8948	2.923	2.989	0.3797
Diabetes Care	1.6899	2.0053	2.082	2.1942	2.4343	2.475	2.658	2.508	0.0004↑
Eur Heart J	1.2961	1.5694	1.7919	2.001	2.3426	2.5218	2.881	3.010	<.0001↑
Eur Resp J	0.9766	1.0266	0.96665	1.035	1.188	1.4831	1.603	1.775	0.0007
Gastroenterology	3.4136	3.634	3.7531	3.6536	3.7979	3.8114	3.869	3.913	0.0018↑
Gut	1.7267	1.7749	1.7387	1.8418	1.9368	2.2264	2.489	2.551	0.0006↑
Hepatology	2.1530	2.3941	2.5945	2.681	2.8068	2.9349	2.981	3.363	<.0001↑
Intens Care Med	0.7029	0.68186	0.82905	0.75332	0.92444	0.92381	1.021	1.214	0.0007↑
J Allergy Clin Immunol	1.4877	1.6964	1.6234	1.7028	1.9319	2.1341	2.074	2.488	0.0004↑
J Am Coll Cardiol	2.7043	2.6455	2.7964	3.0063	3.0826	3.3537	3.803	3.727	0.0002↑
J Bone Miner Res	2.2700	2.2391	2.1552	1.9007	2.2226	2.1188	2.078	2.295	0.8237
J Clin Endocr Metab	1.5449	1.589	1.7449	1.7975	1.8812	1.8724	1.797	1.803	0.0224↑
J Clin Oncol	2.5979	2.8226	3.1852	3.2224	3.3732	3.475	3.916	4.164	<.0001↑
J Infect Dis	1.8863	1.9035	1.7964	1.8621	1.9332	2.049	2.051	2.064	0.0133↑
J Natl Cancer I	5.5065	5.6202	5.6551	5.4014	5.6045	5.4732	5.821	5.791	0.1822
J Neurosci	4.1098	3.9737	3.7925	3.6898	3.6531	3.4425	3.301	3.544	0.0014↓
JAMA	5.8387	6.8129	8.26	9.3565	9.7762	10.29	10.644	11.153	<.0001↑
Lancet	4.6325	4.8334	5.4905	6.6371	7.7322	8.6351	9.318	9.953	<.0001↑
Lancet Infect Dis	n/a§	n/a§	n/a§	5.0478	4.9968	4.9689	4.795	4.689	0.0091↓
Lancet Oncol	n/a§	n/a§	2.1952	2.6062	2.61	2.6211	3.765	3.947	0.0110↑
N Engl J Med	11.571	12.484	13.166	14.399	15.183	16.825	17.864	18.764	<.0001↑
Rheumatology	0.7807	2.0989	1.2218	1.0221	1.162	1.2518	1.237	1.188	0.8238
Thorax	1.3673	1.5481	1.4797	1.6867	1.8039	2.0772	2.098	2.280	<.0001↑

§ n/a  =  not available.

Significant increase over time denoted by (↑), while significant decrease is denoted (↓).

## Discussion

There is no universally accepted metric for assessing the “quality” and “influence” of journals to the scientific community. In the Journal Citation Reports, ISI provides several attributes for assessing quality including total citations, IF, ES, and AIS. Of these the most widely used metric is the IF. However, the major shortcoming of IF is that it is sensitive to the number of original research papers published per year. Because in general review papers and guidelines have a higher citation index than that for original papers, by publishing fewer original papers (and more review papers), journals can increase their IF. Paradoxically, however, because original research is the primary engine for generating new scientific knowledge (or validating existing knowledge), by reducing the publication rate of research articles, journals' influence on the scientific discourse of their discipline may decrease. ES is an attempt to capture the “influence” of medical journals on the scientific discourse generated in their respective fields. The present study indicates that over the past 8 years most medical journals (77% evaluated in this study) have increased their IF. However, 26% of the journals have experienced a paradoxical reduction in their ES during this period of time, associated with a decrease in the number of citable items that were published per year in these journals. Interestingly and provocatively, many journals that fall into this category were those with a very high IF such as *the Lancet, Circulation, American Journal of Respiratory and Critical Care Medicine and the Journal of the American Medical Association*. Notable exceptions in this category were *the New England Journal of Medicine* and the *Journal of Clinical Oncology*, both of which experienced a dramatic increase in their IF without any significant changes in their publication rate of citable items. We also found that there were journals which increased their IF, ES, as well as the number of citable items published per year. These included the *European Heart Journal*, and *Critical Care*. Other journals have increased or maintained their IF without decreasing the number of papers published per year or sacrificing their ES values over time. These data indicate that IF and ES in particular can produce dissimilar results and thus highlight the importance of using multiple rather than just one metric in assessing the performance of journals and the impact and influence they have on their respective fields of study.

Our data are consistent with those of Chew et al [20], who showed that the IF of the seven top-ranked general medical journals rose considerably between 1994 and 2005 but the denominators (i.e. the number of citable items per year) either fell or remained constant. Our data are also consistent with those by Bollen et al, who showed that the concept of scientific impact is multi-dimensional that cannot be adequately captured by IF alone [21] and that both usage and citation based measures are needed to understand the scientific impact of journals. It should also be noted in our analysis that although IF and AIS values are calculated differently, they nonetheless produced similar rank order of journals, suggesting that the weighting system of AIS does not significantly modify the performance status of the journals. The major discrepancies occurred only when the denominator of AIS was removed (yielding ES values), which highlights the importance of quantity of publications in the determination of scientific impact of journals.

There are important implications for these data. Firstly, it is essential that authors take into account not only the IF of journals on deciding where to send their paper but also the ES, as journals with high IF but low ES may have low readership and have little influence on their respective field, although in general papers that are highly accessed and viewed are cited more frequently than those that have limited access [22], [23], [24]. Secondly, IF must be viewed in the context of other metrics such as ES and AIS, which takes into account not only the quantity but also the quality of the citations. Thirdly, the rise of the journal IF over the past decade likely reflects the increase in the citation rate of papers published in these journals. However, it is possible that in some journals, the rise in their IF may in part reflect a reduction in the number of original articles published per year. The potential paradox is that by doing so these journals may be limiting their influence. Thus, as with individual researchers, journals should use IF in conjunction with other metrics such as ES in assessing the relevance and “impact” of their journals in their respective field.

There were limitations to this study. Firstly, ES was used as a surrogate for the “influence” of journals. However, this metric has never been fully validated for this outcome. In the same vein, IF has never been fully validated as measure of “quality”, though it is widely used in this fashion. Secondly, there are other conventional metrics of journal quality such as immediacy index, citation half-life, or PageRank based metrics such as SCImago journal rank indicator[25] that were not considered in the present analysis. Thirdly, an important aspect of understanding the influence of journals is to determine the size and make-up of the readership, which was not done in the present study. Some [22], [23], [24] but not all [26]studies suggest that papers that are viewed more frequently receive higher citation rates than those that are accessed infrequently. Fourthly, we did not determine the reasons for the rise and fall of IF, ES and citable items in these journals. A previous study suggested that the temporal increases in IF for certain journals may reflect several factors including active recruitment of “high-impact” papers by journal editors, acceleration of the review and publication process, early on-line publication of accepted articles, media promotion of articles and journals, and the increase in the number of journals included in the ISI database [20]. The reasons for the fall in the citable items for certain journals are also unclear. Some explanations include journals becoming more selective of the articles that they were accepting, and re-design of journals leading to fewer pages [20]. Whatever the reason, by reducing the citable items, some journals may have (intentionally or unintentionally) increased their IF.

In summary, the present study indicates that IF and ES produce similar rank order of medical journals; however, some important discordances occur. In general, journals that publish a lot of papers have higher ES values than would be expected for their IF. Conversely, journals that publish a small volume of papers have lower ES values than expected for their IF. Some journals have increased their IF and at the same time reduced the number of papers that they publish per year, which may have reduced their influence on the field. Medical journals should carefully balance the important of IF and ES in their editorial composition of the quality and quantity of articles published.

## References

[1] Garfield E (1996). How can impact factors be improved?. BMJ.

[2] Horgan A (2002). BMJ's impact factor increases by 24%.. BMJ.

[3] Parrillo JE (2005). Our Journal, Critical Care Medicine, in 2005: high impact factor, rapid manuscript review, growing submissions, and widespread distribution.. Crit Care Med.

[4] Tobin MJ (2004). Thirty years of impact factor and the Journal.. Am J Respir Crit Care Med.

[5] Wedzicha JA, Johnston SL, Mitchell DM (2005). Journal impact factors for 2004: another rise for Thorax.. Thorax.

[6] Saha S, Saint S, Christakis DA (2003). Impact factor: a valid measure of journal quality?. J Med Libr Assoc.

[7] Seglen PO (1997). Why the impact factor of journals should not be used for evaluating research.. BMJ.

[8] Favaloro EJ (2008). Measuring the quality of journals and journal articles: the impact factor tells but a portion of the story.. Semin Thromb Hemost.

[9] Weale AR, Bailey M, Lear PA (2004). The level of non-citation of articles within a journal as a measure of quality: a comparison to the impact factor.. BMC Med Res Methodol.

[10] Callaham M, Wears RL, Weber E (2002). Journal prestige, publication bias, and other characteristics associated with citation of published studies in peer-reviewed journals.. JAMA.

[11] Marashi SA (2005). On the identity of “citers”: are papers promptly recognized by other investigators?. Med Hypotheses.

[12] Bergstrom CT, West JD, Wiseman MA (2008). The Eigenfactor metrics.. J Neurosci.

[13] Bollen J, Rodriguez M, Van de Sompel H (2006). Journal status.. Scientometrics.

[14] Dellavalle RP, Schilling LM, Rodriguez MA, Van de Sompel H, Bollen J (2007). Refining dermatology journal impact factors using PageRank.. J Am Acad Dermatol.

[15] Pinski G, Narin F (1976). Citation influence for journal aggregates of scientific publications: Theory, with application to the literature of physics.. Information Processing and Management.

[16] Davis PM (2008). Eigenfactor: does the principle of repeated improvement result in better estimates than raw citation counts?. Journal of the American Society for Information Science and Technology.

[17] Fersht A (2009). The most influential journals: Impact Factor and Eigenfactor.. Proc Natl Acad Sci U S A.

[18] Chen P, Xie H, Maslov S, Redner S (2007). Finding scientific gems with Google's PageRank algorithm.. Journal of Informetrics.

[19] Hakkalamani S, Rawal A, Hennessy MS, Parkinson RW (2006). The impact factor of seven orthopaedic journals: factors influencing it.. J Bone Joint Surg Br.

[20] Chew M, Villanueva EV, Van Der Weyden MB (2007). Life and times of the impact factor: retrospective analysis of trends for seven medical journals (1994-2005) and their Editors' views.. J R Soc Med.

[21] Bollen J, Van de Sompel H, Hagberg A, Chute R (2009). A principal component analysis of 39 scientific impact measures.. PLoS One.

[22] Brody T, Harnad S, Carr L (2006). Earlier Web usage statistics as predictors of later citation impact.. Journal of the American Society for Information Science and Technology.

[23] Eysenbach G (2006). Citation advantage of open access articles.. PLoS Biol.

[24] Perneger TV (2004). Relation between online “hit counts” and subsequent citations: prospective study of research papers in the BMJ.. BMJ.

[25] Falagas ME, Kouranos VD, Arencibia-Jorge R, Karageorgopoulos DE (2008). Comparison of SCImago journal rank indicator with journal impact factor.. FASEB J.

[26] Davis PM, Lewenstein BV, Simon DH, Booth JG, Connolly MJ (2008). Open access publishing, article downloads, and citations: randomised controlled trial.. BMJ.

